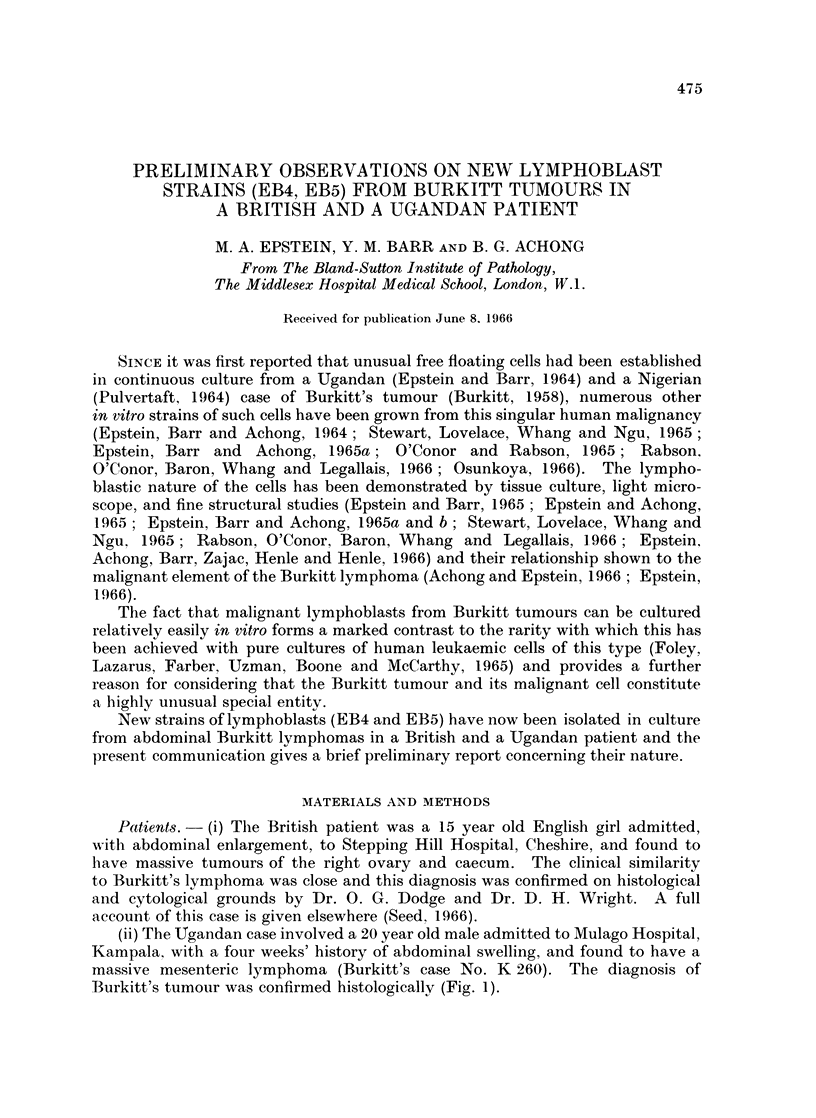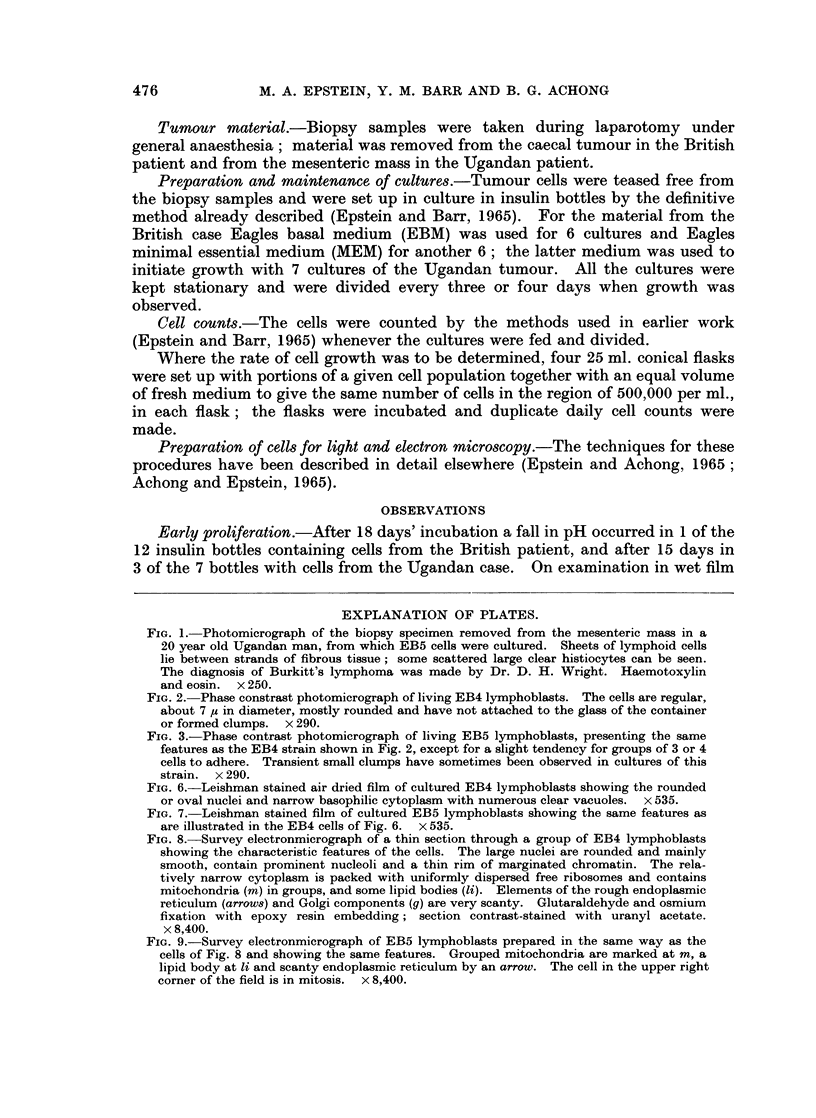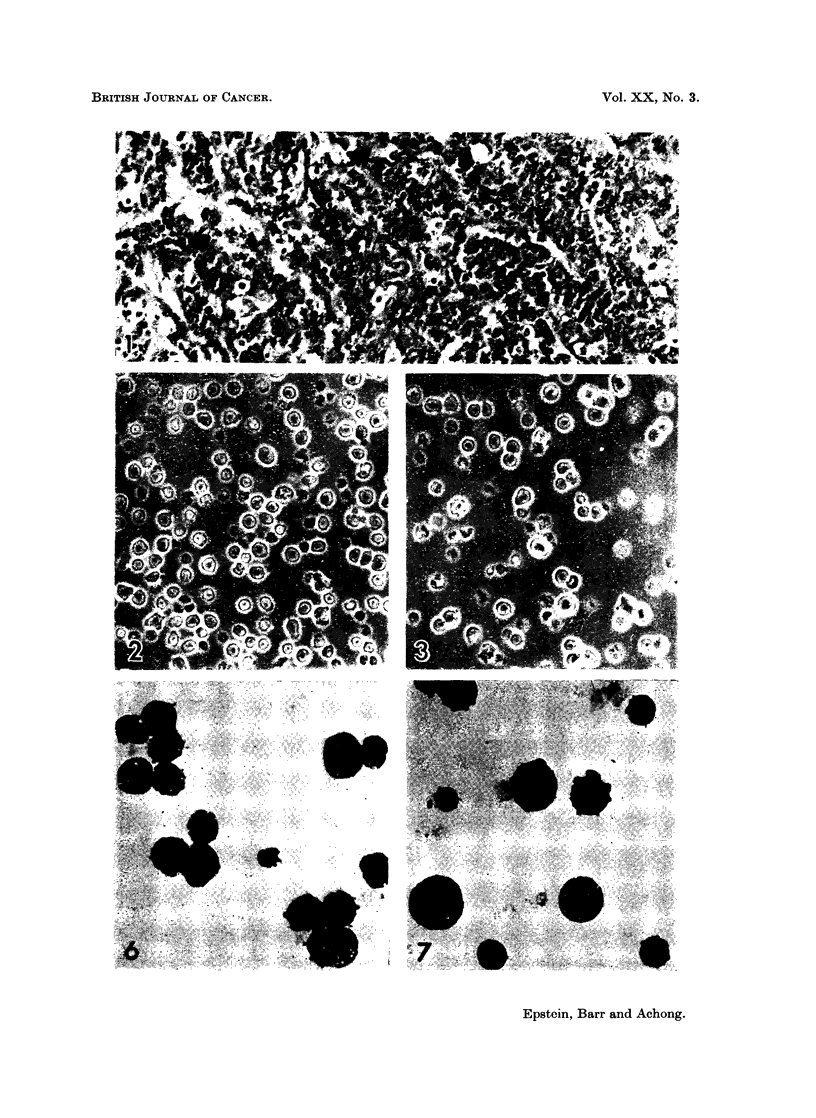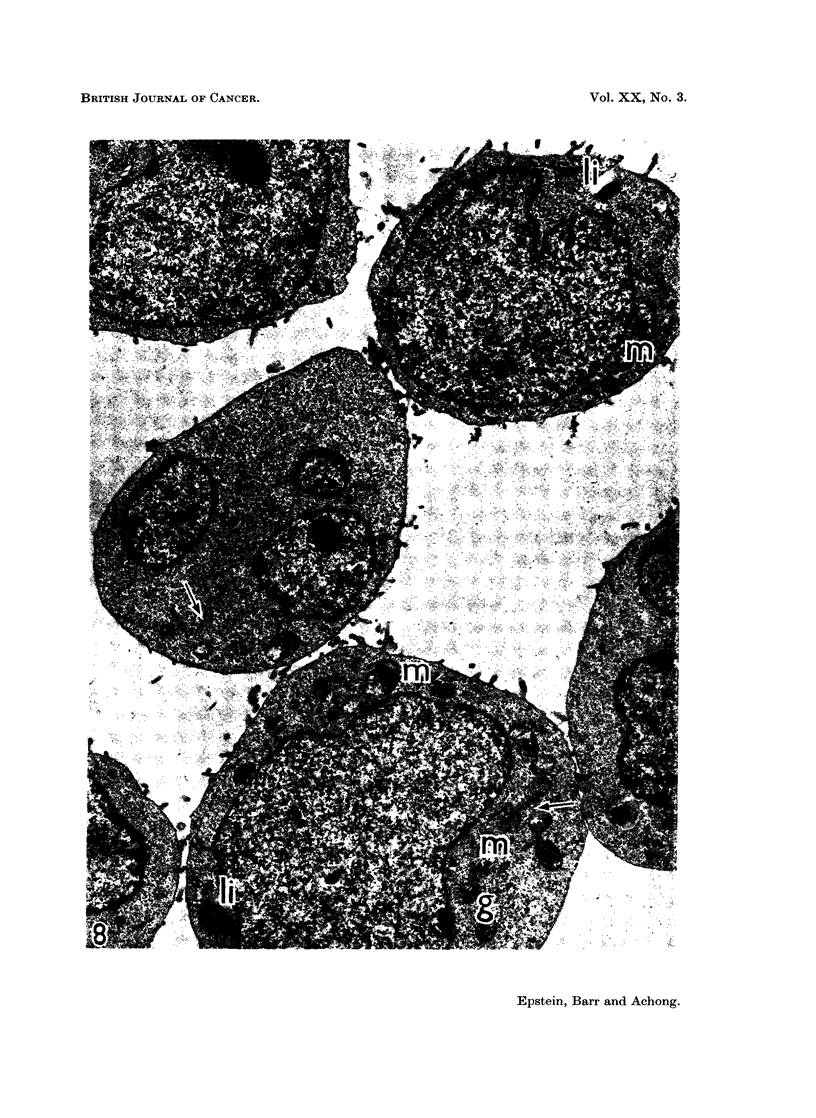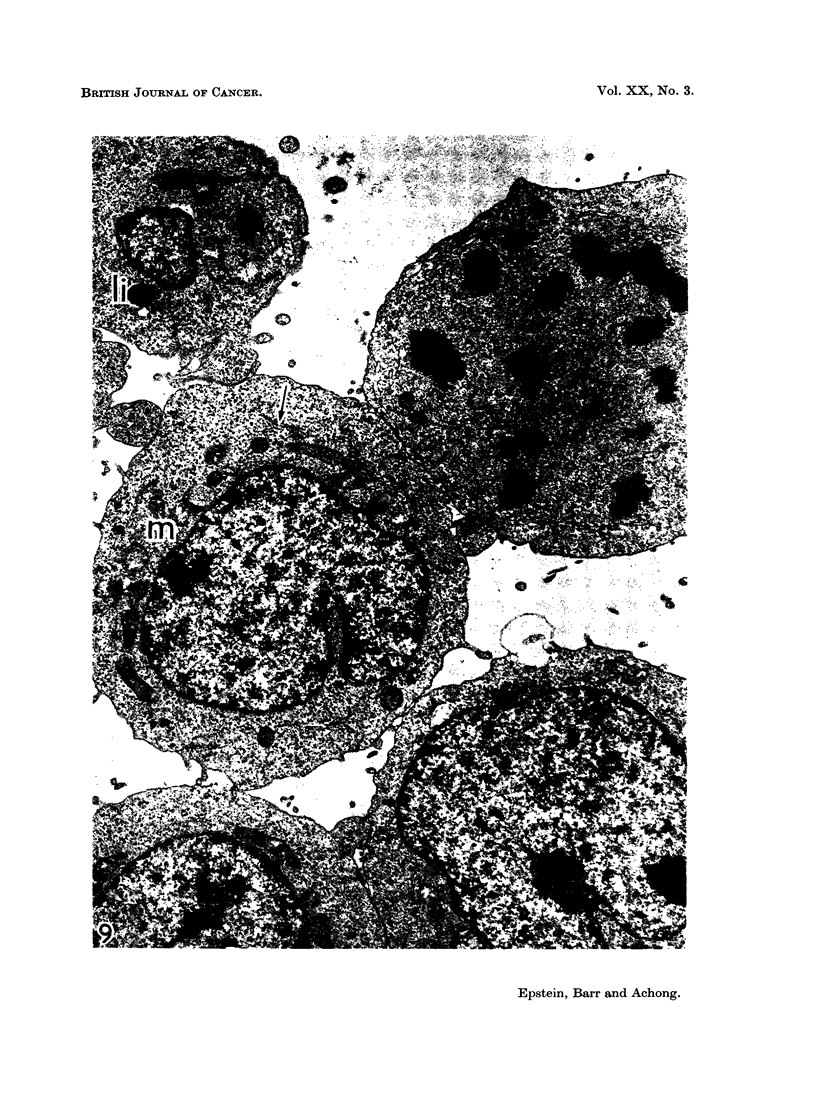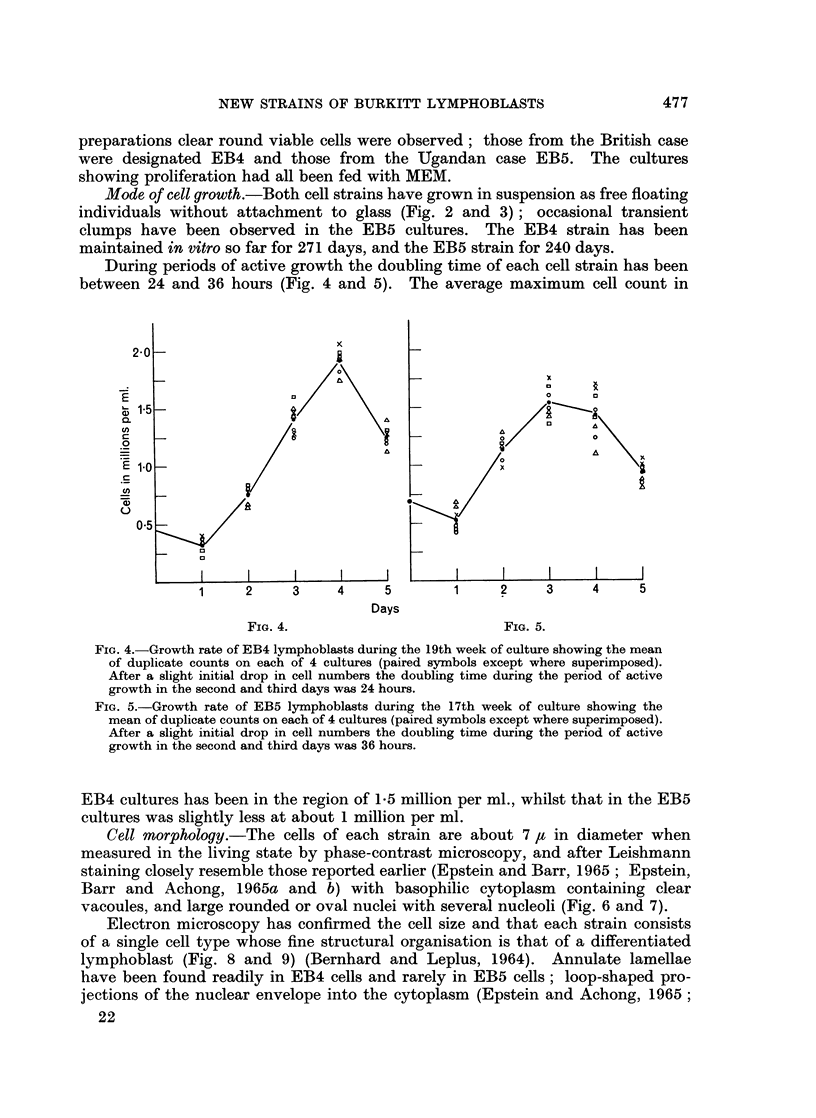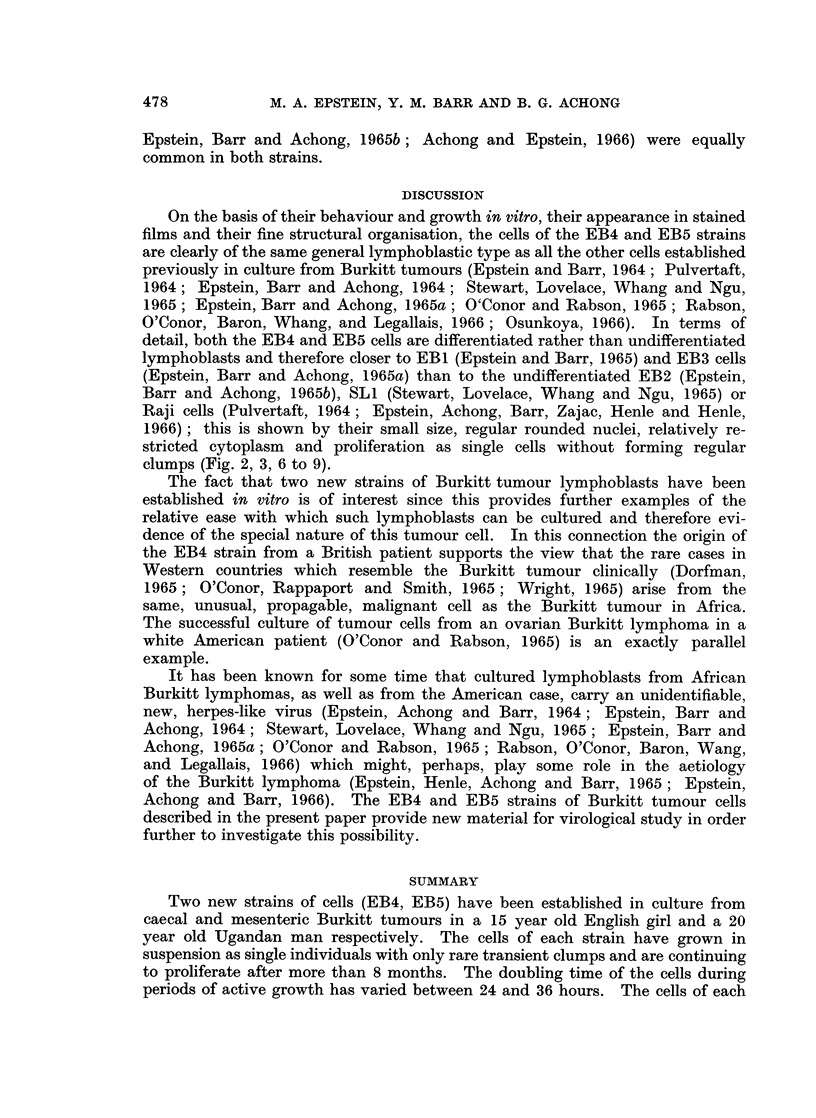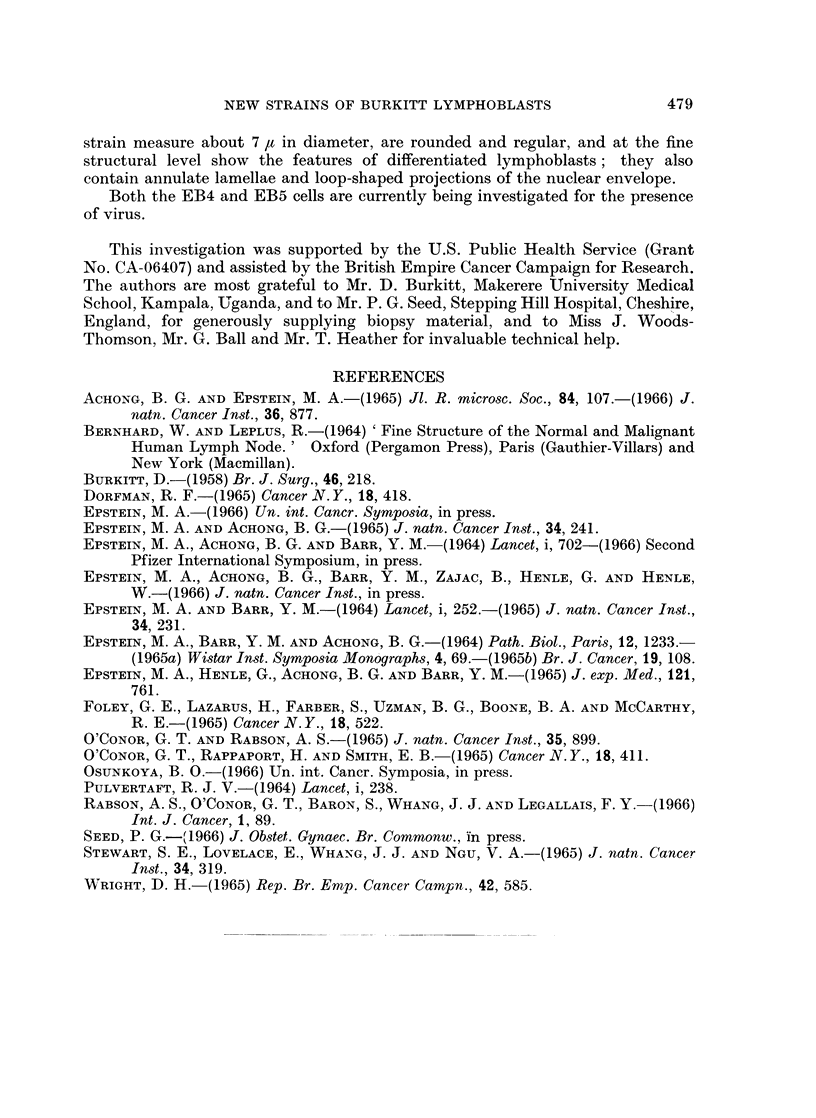# Preliminary observations on new lymphoblast strains (EB4, EB5) from Burkitt tumours in a British and a Ugandan patient.

**DOI:** 10.1038/bjc.1966.58

**Published:** 1966-09

**Authors:** M. A. Epstein, Y. M. Barr, B. G. Achong

## Abstract

**Images:**


### PRELIMINARY OBSERVATIONS ON NEW LYMPHOBLAST STRAINS (EB4, EB5) FROM BURKITT TUMOURS IN A BRITISH AND A UGANDAN PATIENT

M. A. EPSTEIN, Y. M. BARR AND B. G. ACHONG

*From The Bland-Sutton Institute of Pathology, The Middlesex Hospital Medical School, London, W.1.*

Received for publication June 8, 1966

SINCE it was first reported that unusual free floating cells had been established in continuous culture from a Ugandan (Epstein and Barr, 1964) and a Nigerian (Pulvertaft, 1964) case of Burkitt's tumour (Burkitt, 1958), numerous other *in vitro* strains of such cells have been grown from this singular human malignancy (Epstein, Barr and Achong, 1964; Stewart, Lovelace, Whang and Ngu, 1965; Epstein, Barr and Achong, 1965*a*; O'Conor and Rabson, 1965; Rabson, O'Conor, Baron, Whang and Legallais, 1966; Osunkoya, 1966). The lymphoblastic nature of the cells has been demonstrated by tissue culture, light microscope, and fine structural studies (Epstein and Barr, 1965; Epstein and Achong, 1965; Epstein, Barr and Achong, 1965*a* and *b*; Stewart, Lovelace, Whang and Ngu, 1965; Rabson, O'Conor, Baron, Whang and Legallais, 1966; Epstein, Achong, Barr, Zajac, Henle and Henle, 1966) and their relationship shown to the malignant element of the Burkitt lymphoma (Achong and Epstein, 1966; Epstein, 1966).

The fact that malignant lymphoblasts from Burkitt tumours can be cultured relatively easily *in vitro* forms a marked contrast to the rarity with which this has been achieved with pure cultures of human leukaemic cells of this type (Foley, Lazarus, Farber, Uzman, Boone and McCarthy, 1965) and provides a further reason for considering that the Burkitt tumour and its malignant cell constitute a highly unusual special entity.

New strains of lymphoblasts (EB4 and EB5) have now been isolated in culture from abdominal Burkitt lymphomas in a British and a Ugandan patient and the present communication gives a brief preliminary report concerning their nature.

### MATERIALS AND METHODS

*Patients.* — (i) The British patient was a 15 year old English girl admitted, with abdominal enlargement, to Stepping Hill Hospital, Cheshire, and found to have massive tumours of the right ovary and caecum. The clinical similarity to Burkitt's lymphoma was close and this diagnosis was confirmed on histological and cytological grounds by Dr. O. G. Dodge and Dr. D. H. Wright. A full account of this case is given elsewhere (Seed, 1966).

(ii) The Ugandan case involved a 20 year old male admitted to Mulago Hospital, Kampala, with a four weeks' history of abdominal swelling, and found to have a massive mesenteric lymphoma (Burkitt's case No. K 260). The diagnosis of Burkitt's tumour was confirmed histologically (Fig. 1).

*Tumour material.*—Biopsy samples were taken during laparotomy under general anaesthesia ; material was removed from the caecal tumour in the British patient and from the mesenteric mass in the Ugandan patient.

*Preparation and maintenance of cultures.*—Tumour cells were teased free from the biopsy samples and were set up in culture in insulin bottles by the definitive method already described (Epstein and Barr, 1965). For the material from the British case Eagles basal medium (EBM) was used for 6 cultures and Eagles minimal essential medium (MEM) for another 6 ; the latter medium was used to initiate growth with 7 cultures of the Ugandan tumour. All the cultures were kept stationary and were divided every three or four days when growth was observed.

*Cell counts.*—The cells were counted by the methods used in earlier work (Epstein and Barr, 1965) whenever the cultures were fed and divided.

Where the rate of cell growth was to be determined, four 25 ml. conical flasks were set up with portions of a given cell population together with an equal volume of fresh medium to give the same number of cells in the region of 500,000 per ml., in each flask ; the flasks were incubated and duplicate daily cell counts were made.

*Preparation of cells for light and electron microscopy.*—The techniques for these procedures have been described in detail elsewhere (Epstein and Achong, 1965 ; Achong and Epstein, 1965).

### OBSERVATIONS

*Early proliferation.*—After 18 days' incubation a fall in pH occurred in 1 of the 12 insulin bottles containing cells from the British patient, and after 15 days in 3 of the 7 bottles with cells from the Ugandan case. On examination in wet film

---

### EXPLANATION OF PLATES.

FIG. 1.—Photomicrograph of the biopsy specimen removed from the mesenteric mass in a 20 year old Ugandan man, from which EB5 cells were cultured. Sheets of lymphoid cells lie between strands of fibrous tissue ; some scattered large clear histiocytes can be seen. The diagnosis of Burkitt's lymphoma was made by Dr. D. H. Wright. Haemotoxylin and eosin. ×250.

FIG. 2.—Phase constrast photomicrograph of living EB4 lymphoblasts. The cells are regular, about 7 $\mu$ in diameter, mostly rounded and have not attached to the glass of the container or formed clumps. ×290.

FIG. 3.—Phase contrast photomicrograph of living EB5 lymphoblasts, presenting the same features as the EB4 strain shown in Fig. 2, except for a slight tendency for groups of 3 or 4 cells to adhere. Transient small clumps have sometimes been observed in cultures of this strain. ×290.

FIG. 6.—Leishman stained air dried film of cultured EB4 lymphoblasts showing the rounded or oval nuclei and narrow basophilic cytoplasm with numerous clear vacuoles. ×535.

FIG. 7.—Leishman stained film of cultured EB5 lymphoblasts showing the same features as are illustrated in the EB4 cells of Fig. 6. ×535.

FIG. 8.—Survey electronmicrograph of a thin section through a group of EB4 lymphoblasts showing the characteristic features of the cells. The large nuclei are rounded and mainly smooth, contain prominent nucleoli and a thin rim of marginated chromatin. The relatively narrow cytoplasm is packed with uniformly dispersed free ribosomes and contains mitochondria (*m*) in groups, and some lipid bodies (*li*). Elements of the rough endoplasmic reticulum (*arrows*) and Golgi components (*g*) are very scanty. Glutaraldehyde and osmium fixation with epoxy resin embedding ; section contrast-stained with uranyl acetate. ×8,400.

FIG. 9.—Survey electronmicrograph of EB5 lymphoblasts prepared in the same way as the cells of Fig. 8 and showing the same features. Grouped mitochondria are marked at *m*, a lipid body at *li* and scanty endoplasmic reticulum by an *arrow*. The cell in the upper right corner of the field is in mitosis. ×8,400.

1

2

3

6

7

li
m
m
m
li
g
8

Epstein, Barr and Achong.

preparations clear round viable cells were observed ; those from the British case were designated EB4 and those from the Ugandan case EB5. The cultures showing proliferation had all been fed with MEM.

*Mode of cell growth.*—Both cell strains have grown in suspension as free floating individuals without attachment to glass (Fig. 2 and 3) ; occasional transient clumps have been observed in the EB5 cultures. The EB4 strain has been maintained *in vitro* so far for 271 days, and the EB5 strain for 240 days.

During periods of active growth the doubling time of each cell strain has been between 24 and 36 hours (Fig. 4 and 5). The average maximum cell count in

FIG. 4. FIG. 5.

FIG. 4.—Growth rate of EB4 lymphoblasts during the 19th week of culture showing the mean of duplicate counts on each of 4 cultures (paired symbols except where superimposed). After a slight initial drop in cell numbers the doubling time during the period of active growth in the second and third days was 24 hours.

FIG. 5.—Growth rate of EB5 lymphoblasts during the 17th week of culture showing the mean of duplicate counts on each of 4 cultures (paired symbols except where superimposed). After a slight initial drop in cell numbers the doubling time during the period of active growth in the second and third days was 36 hours.

EB4 cultures has been in the region of 1·5 million per ml., whilst that in the EB5 cultures was slightly less at about 1 million per ml.

*Cell morphology.*—The cells of each strain are about 7 $\mu$ in diameter when measured in the living state by phase-contrast microscopy, and after Leishmann staining closely resemble those reported earlier (Epstein and Barr, 1965 ; Epstein, Barr and Achong, 1965*a* and *b*) with basophilic cytoplasm containing clear vacoules, and large rounded or oval nuclei with several nucleoli (Fig. 6 and 7).

Electron microscopy has confirmed the cell size and that each strain consists of a single cell type whose fine structural organisation is that of a differentiated lymphoblast (Fig. 8 and 9) (Bernhard and Leplus, 1964). Annulate lamellae have been found readily in EB4 cells and rarely in EB5 cells ; loop-shaped projections of the nuclear envelope into the cytoplasm (Epstein and Achong, 1965 ;

Epstein, Barr and Achong, 1965*b* ; Achong and Epstein, 1966) were equally common in both strains.

### DISCUSSION

On the basis of their behaviour and growth *in vitro*, their appearance in stained films and their fine structural organisation, the cells of the EB4 and EB5 strains are clearly of the same general lymphoblastic type as all the other cells established previously in culture from Burkitt tumours (Epstein and Barr, 1964 ; Pulvertaft, 1964 ; Epstein, Barr and Achong, 1964 ; Stewart, Lovelace, Whang and Ngu, 1965 ; Epstein, Barr and Achong, 1965*a* ; O'Conor and Rabson, 1965 ; Rabson, O'Conor, Baron, Whang, and Legallais, 1966 ; Osunkoya, 1966). In terms of detail, both the EB4 and EB5 cells are differentiated rather than undifferentiated lymphoblasts and therefore closer to EB1 (Epstein and Barr, 1965) and EB3 cells (Epstein, Barr and Achong, 1965*a*) than to the undifferentiated EB2 (Epstein, Barr and Achong, 1965*b*), SL1 (Stewart, Lovelace, Whang and Ngu, 1965) or Raji cells (Pulvertaft, 1964 ; Epstein, Achong, Barr, Zajac, Henle and Henle, 1966) ; this is shown by their small size, regular rounded nuclei, relatively restricted cytoplasm and proliferation as single cells without forming regular clumps (Fig. 2, 3, 6 to 9).

The fact that two new strains of Burkitt tumour lymphoblasts have been established *in vitro* is of interest since this provides further examples of the relative ease with which such lymphoblasts can be cultured and therefore evidence of the special nature of this tumour cell. In this connection the origin of the EB4 strain from a British patient supports the view that the rare cases in Western countries which resemble the Burkitt tumour clinically (Dorfman, 1965 ; O'Conor, Rappaport and Smith, 1965 ; Wright, 1965) arise from the same, unusual, propagable, malignant cell as the Burkitt tumour in Africa. The successful culture of tumour cells from an ovarian Burkitt lymphoma in a white American patient (O'Conor and Rabson, 1965) is an exactly parallel example.

It has been known for some time that cultured lymphoblasts from African Burkitt lymphomas, as well as from the American case, carry an unidentifiable, new, herpes-like virus (Epstein, Achong and Barr, 1964 ; Epstein, Barr and Achong, 1964 ; Stewart, Lovelace, Whang and Ngu, 1965 ; Epstein, Barr and Achong, 1965*a* ; O'Conor and Rabson, 1965 ; Rabson, O'Conor, Baron, Wang, and Legallais, 1966) which might, perhaps, play some role in the aetiology of the Burkitt lymphoma (Epstein, Henle, Achong and Barr, 1965 ; Epstein, Achong and Barr, 1966). The EB4 and EB5 strains of Burkitt tumour cells described in the present paper provide new material for virological study in order further to investigate this possibility.

### SUMMARY

Two new strains of cells (EB4, EB5) have been established in culture from caecal and mesenteric Burkitt tumours in a 15 year old English girl and a 20 year old Ugandan man respectively. The cells of each strain have grown in suspension as single individuals with only rare transient clumps and are continuing to proliferate after more than 8 months. The doubling time of the cells during periods of active growth has varied between 24 and 36 hours. The cells of each strain measure about 7 $\mu$ in diameter, are rounded and regular, and at the fine structural level show the features of differentiated lymphoblasts ; they also contain annulate lamellae and loop-shaped projections of the nuclear envelope.

Both the EB4 and EB5 cells are currently being investigated for the presence of virus.

This investigation was supported by the U.S. Public Health Service (Grant No. CA-06407) and assisted by the British Empire Cancer Campaign for Research. The authors are most grateful to Mr. D. Burkitt, Makerere University Medical School, Kampala, Uganda, and to Mr. P. G. Seed, Stepping Hill Hospital, Cheshire, England, for generously supplying biopsy material, and to Miss J. Woods-Thomson, Mr. G. Ball and Mr. T. Heather for invaluable technical help.

### REFERENCES

ACHONG, B. G. AND EPSTEIN, M. A.—(1965) *Jl. R. microsc. Soc.*, **84**, 107.—(1966) *J. natn. Cancer Inst.*, **36**, 877.
BERNHARD, W. AND LEPLUS, R.—(1964) ' Fine Structure of the Normal and Malignant Human Lymph Node. ' Oxford (Pergamon Press), Paris (Gauthier-Villars) and New York (Macmillan).
BURKITT, D.—(1958) *Br. J. Surg.*, **46**, 218.
DORFMAN, R. F.—(1965) *Cancer N.Y.*, **18**, 418.
EPSTEIN, M. A.—(1966) *Un. int. Cancr. Symposia*, in press.
EPSTEIN, M. A. AND ACHONG, B. G.—(1965) *J. natn. Cancer Inst.*, **34**, 241.
EPSTEIN, M. A., ACHONG, B. G. AND BARR, Y. M.—(1964) *Lancet*, i, 702—(1966) Second Pfizer International Symposium, in press.
EPSTEIN, M. A., ACHONG, B. G., BARR, Y. M., ZAJAC, B., HENLE, G. AND HENLE, W.—(1966) *J. natn. Cancer Inst.*, in press.
EPSTEIN, M. A. AND BARR, Y. M.—(1964) *Lancet*, i, 252.—(1965) *J. natn. Cancer Inst.*, **34**, 231.
EPSTEIN, M. A., BARR, Y. M. AND ACHONG, B. G.—(1964) *Path. Biol., Paris*, **12**, 1233.—(1965*a*) *Wistar Inst. Symposia Monographs*, **4**, 69.—(1965*b*) *Br. J. Cancer*, **19**, 108.
EPSTEIN, M. A., HENLE, G., ACHONG, B. G. AND BARR, Y. M.—(1965) *J. exp. Med.*, **121**, 761.
FOLEY, G. E., LAZARUS, H., FARBER, S., UZMAN, B. G., BOONE, B. A. AND McCARTHY, R. E.—(1965) *Cancer N.Y.*, **18**, 522.
O'CONOR, G. T. AND RABSON, A. S.—(1965) *J. natn. Cancer Inst.*, **35**, 899.
O'CONOR, G. T., RAPPAPORT, H. AND SMITH, E. B.—(1965) *Cancer N.Y.*, **18**, 411.
OSUNKOYA, B. O.—(1966) Un. int. Cancr. Symposia, in press.
PULVERTAFT, R. J. V.—(1964) *Lancet*, i, 238.
RABSON, A. S., O'CONOR, G. T., BARON, S., WHANG, J. J. AND LEGALLAIS, F. Y.—(1966) *Int. J. Cancer*, **1**, 89.
SEED, P. G.—(1966) *J. Obstet. Gynaec. Br. Commonw.*, in press.
STEWART, S. E., LOVELACE, E., WHANG, J. J. AND NGU, V. A.—(1965) *J. natn. Cancer Inst.*, **34**, 319.
WRIGHT, D. H.—(1965) *Rep. Br. Emp. Cancer Campn.*, **42**, 585.